# Hepatocellular Carcinoma With Bile Duct Tumor Thrombus: A Clinicopathological Analysis of Factors Predictive of Recurrence and Outcome After Surgery

**DOI:** 10.1097/MD.0000000000000364

**Published:** 2015-01-09

**Authors:** Hong Zeng, Lei-bo Xu, Jian-ming Wen, Rui Zhang, Man-sheng Zhu, Xiang-de Shi, Chao Liu

**Affiliations:** From the Guangdong Provincial Key Laboratory of Malignant Tumor Epigenetics and Gene Regulation (HZ, L-bX, RZ, M-sZ, X-dS, CL), Medical Research Center; Department of Pathology (HZ); Department of Hepato-pancreato-biliary Surgery (L-bX, RZ, M-sZ, X-dS, CL), Sun Yat-sen Memorial Hospital; and Department of Pathology (J-mW), First Affiliated Hospital, Sun Yat-sen University, Guangzhou, China.

## Abstract

Although hepatocellular carcinoma (HCC) with bile duct tumor thrombus (BDTT) is a rare entity, most patients experience tumor recurrence even after curative resection and the prognosis remains dismal. This study aimed to analyze the clinicopathological risk factors for recurrence and poor outcome after surgical treatment of HCC with BDTT.

Clinicopathological data of 37 patients with HCC and BDTT who underwent surgical treatment from July 2005 to June 2012 at the authors’ hospital were reviewed retrospectively. Prognostic factors and potential risk factors for recurrence were assessed by Cox proportional hazard model and binary logistic regression model, respectively.

Among the 37 patients, anatomical and nonanatomical liver resection was performed in 26 and 11 patients, respectively. The resection was considered curative in 19 patients and palliative in 18 patients. Also, 21 cases had tumor recurrence after operation and 7 cases of them were reoperated. Multivariate binary logistic regression model revealed that surgical curability was the only independent risk factor associated with postoperative tumor recurrence (*P* = 0.034). In addition, postoperative overall survival rates at 1, 2, and 3 years were 64.2%, 38.9%, and 24.3%, respectively. Cox multivariate analysis indicated that surgical curability and tumor recurrence were independent prognostic factors for both overall survival and recurrence-free survival (*P* < 0.05).

Although patients with HCC and BDTT had a relatively high rate of early recurrence after surgery, relatively favorable long-term outcome after curative hepatic resection could be achieved. Therefore, extensive and curative surgical treatment should be recommended when complete resection can be achieved and liver functional reserve is satisfactory.

## INTRODUCTION

Hepatocellular carcinoma (HCC) is one of the most fatal malignancies worldwide. The incidence continues to increase over the past decades and the long-term prognosis remains dismal even after radical surgery or liver transplantation.^1,2^ HCC with bile duct tumor thrombus (BDTT) is a rare entity and only accounts for 1.2 to 12.9% of HCC. It was often misdiagnosed as biliary calculi, cholangiocarcinoma, or liver failure, of advanced HCC for similar clinical presentations and imaging characteristics. Due to advances in imaging and increased understanding of this entity, more and more patients with HCC and BDTT were confirmed preoperatively. Complete resection is regarded as the only way that possibly cures the patients.^3–6^ However, a patient with HCC and BDTT has a relatively high risk of early recurrence, resulting in poor prognosis even after curative hepatic resection or liver transplantation.^7–12^

Although several studies have explored the relationships between various clinicopathological features and the outcome of patients with HCC and BDTT, it is currently unknown whether these patients could benefit substantially from surgery. Moreover, no generally accepted standard of curative hepatic resection is available until now.^6–11^ In this study, therefore, we retrospectively studied the clinicopathological features of 37 patients with HCC and BDTT who underwent surgical treatment at our hospital, and aimed to identify the predictive factors for recurrence and poor prognosis after surgery. In addition, the therapeutic effects of different surgical treatment modalities based on histological discoveries were discussed.

## PATIENTS AND METHODS

### Patients and Definitions

From July 2005 to June 2012, 1030 patients with HCC underwent surgery at the Department of Hepato-pancreato-biliary Surgery, Sun Yat-sen Memorial Hospital, Sun Yat-sen University. The diagnosis was confirmed histologically after the operation for all patients. BDTT was identified histopathologically in 37 patients. The data of clinicopathological features and prognoses of these 37 patients were collected and analyzed retrospectively. The project was approved by the ethical committee of the hospital and in accordance with the Helsinki Declaration of 1975. All written informed consents were obtained from the patients or their guardians.

Tumor stage was classified in accordance with the criteria of the American Joint Committee on Cancer (AJCC).^13^ Histological grade was estimated according to the Edmondson–Steiner classification.^14^ BDTT was classified into 2 categories according to the following criteria: microscopic BDTT, which represents that the tumor thrombus was found in more than second branch of the biliary tree, and macroscopic BDTT, which represents that tumor thrombus can be seen in no more than the second-order branch.^15^ Macroscopic BDTT can be further classified into type I, type II, and type III according to the classification of Satoh et al.^3^ In these patients, tumor recurrences were defined as reappearance of macro-BDTT or new lesions on imaging during follow-up, and classified into early (≤1 year) and late (>1 year) recurrences as described by Poon et al.^16^

There were 33 men (89.2%) and 4 women (10.8%). The age ranged from 23 to 78 years, and the median age was 50 years. Hepatitis B surface antigen (HBsAg) and liver cirrhosis was detected in 29 patients (78.4%) and 33 patients (89.2%), respectively. High α-fetoprotein (AFP) serum levels (>400 ng/mL) have been found in 32.4% (12/37) of patients. The common symptoms were obstructive jaundice and upper abdominal pain.

### Surgical Modality and Treatment of Recurrence

For the primary tumor, hepatic resection, concurrent bile duct resection, and/or thrombectomy were performed according to tumor characteristics, BDTT location, and hepatic functional reserve. When tumors recurred, patients were treated with repeated hepatectomy and/or thrombectomy whenever possible, or by radio-frequency ablation (RFA), percutaneous ethanol injection therapy (PEI), or transcatheter chemoembolization arterial chemotherapy (TACE) as appropriate.

According to the Couinaud's nomenclature for liver segmentation, anatomical liver resection (AR) was defined as the complete removal of at least 1 entire Couinaud segment (including subsegmentectomy, segmentectomy, sectionectomy, hemihepatectomy, and extended hemihepatectomy); nonanatomical liver resection (NAR) was defined as a limited resection or tumor enucleation regardless of the Couinaud segment.^17–20^ In addition, hepatic resection was considered curative only when the margin was free at histology (bile duct and specimen margins negative for tumor), and serum AFP level and the postoperative radiographic examination (computed tomography scan and ultrasonography) showed no evidence of tumor at 3 months after surgery.^21–24^

### Follow-Up

During the first 6 months after surgery, follow-up visits are scheduled every 1 to 2 months. After that, follow-up visits are scheduled every 3 to 6 months. At each follow-up visit, clinical, laboratory, and radiological (abdominal ultrasound, computed tomography scan, and chest x-ray) data were collected. During the follow-up period, a total of 34 (91.9%) patients were followed up until the end of December 2013 or death, whereas 2 (5.4%) patients died of multiorgan dysfunction syndrome perioperatively and 3 (8.1%) patients were lost.

### Statistical Analysis

Statistical analysis was conducted with the SPSS software package (version 17.0; SPSS Inc, Chicago, IL). Quantitative data were presented as mean ± standard error (SE). Prognostic factors were assessed by univariate and multivariate Cox proportional hazard model. Potential risk factors for recurrence were assessed by univariate and multivariate binary logistic regression model. The Kaplan–Meier method (with the log-rank test) was adopted for predicting recurrence and evaluating survival. For all tests, a *P*-value <0.05 was considered statistically significant.

## RESULTS

### Patient Profiles

Among the 37 patients, 25 (67.6%) patients were accompanied by vascular invasion, 5 (13.5%) patients had lymph node metastasis, 1 (2.7%) patient had extrahepatic metastasis, and all of the patients had intact tumor capsules. The mean diameter of the primary tumor was 4.97 ± 0.49 cm (range: 0.5–11 cm). In addition, 9 (24.4%) patients were classified as stage I, 8 (21.6%) as stage II, 14 (37.8%) as stage III, and 6 (16.2%) as stage IV. Tumors were classified as histological grade I in 13.5% (5/37), grade II in 24.3% (9/37), grade III in 51.4% (19/37), and grade IV in 10.8% (4/37) of cases.

### Histopathological Findings of BDTT

**FIGURE 1:**
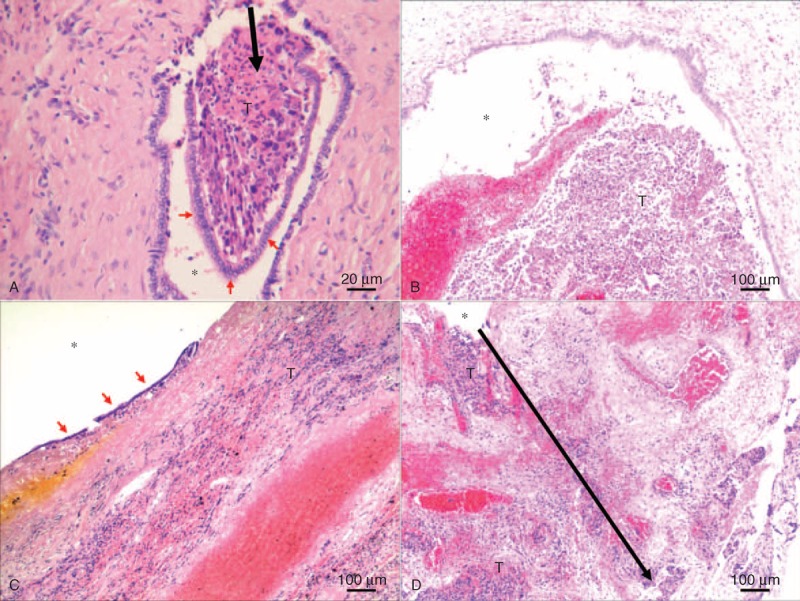
Histopathological findings of BDTT. (A) Tumor thrombus (black arrow) herniated from subepithelium to the lumen by a stalk, being covered with bile duct epithelial layer (red arrow). (B) Hemorrhagic necrosis of tumor thrombus was observed in the lumen of bile duct. It did not adhere to the bile duct wall around it, and no epithelium was found on its surface. (C) Bile duct epithelial fragments (red arrow) were found on the surface of tumor thrombus. (D) Tumor cells were detected in the excised bile duct wall (black arrow). ∗ = Lumen of bile duct. BDTT = bile duct tumor thrombus, T = tumor thrombus.

In this study, 7 patients had microscopic BDTT and 30 patients had macroscopic BDTT (7 patients with type I and 23 patients with type II). Macroscopically, BDTT often appeared as grayish white or brownish green polypoid masses with smooth surface. It tends to grow continuously from peripheral small bile duct to portal large bile duct. In most cases, tumor tissues were found located at subepithelium of bile duct. They herniated from subepithelium to the lumen by a stalk, being covered with intact or fragmentary bile duct epithelial layer (Figure 1A). Only in a small fraction of patients, fragmented or necrotic tumor tissue was found in the lumen of bile duct adjacent to porta hepatis. It did not adhere to the bile duct wall around it, and no epithelium was found on its surface (Figure 1B). In 10 of 16 patients (62.5%) who underwent hepatectomy and thrombectomy, bile duct epithelial fragments were found on the surface of thrombi (Figure 1C), while in 10 of 14 patients (71.4%) who underwent concurrent bile duct resection, tumor cells were detected in the excised large bile duct wall (Figure 1D).

### Surgical Treatment Profiles and Management of Recurrence

**FIGURE 2:**
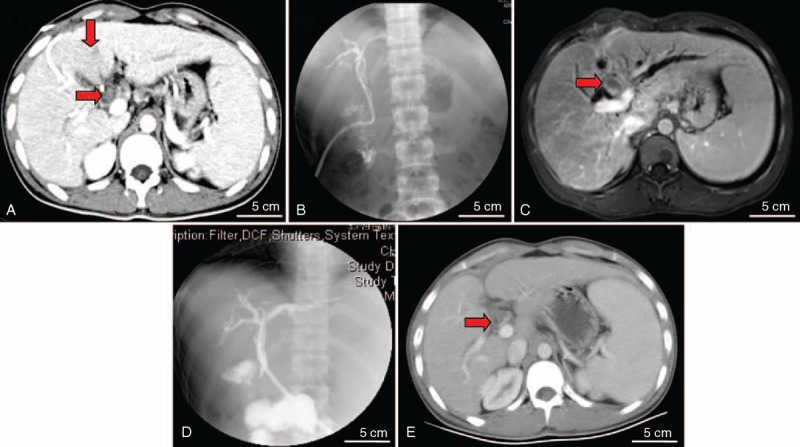
Image data of the patient who experienced BDTT re-recurrence after surgery. (A) HCC at segment 4 (vertical arrow) and tumor thrombus in common hepatic duct (horizontal arrow) were found with computed tomography. (B) Cholangiography showed that no definite filling defect was seen in the biliary system after hepatectomy and thrombectomy. (C) BDTT recurred (horizontal arrow) 7 months after first surgery (observed with functional magnetic resonance imaging). (D) Cholangiography showed that the recurrent BDTT was removed completely by reoperation. (E) Eight months after reoperation, computed tomography revealed that BDTT recurred (horizontal arrow) for the second time. BDTT = bile duct tumor thrombus, HCC = hepatocellular carcinoma.

In the present study, 7 patients with microscopic BDTT were treated by hepatectomy, 14 patients with macroscopic BDTT were treated by hepatectomy plus extrahepatic bile duct resection and hepatojejunostomy, and 16 patients with macroscopic BDTT were treated by hepatectomy plus thrombectomy and T-tube drainage. For the primary tumor, AR and NAR were performed in 26 (70.3%) and 11 (29.7%) patients, respectively. The resection was considered curative in 19 patients (51.4%) and palliative in 18 patients (48.6%) based on the above criteria. A total of 21 patients experienced tumor recurrence after surgery (56.8%), and more than three-quarters (76.2%) recurred within the first 1 year after surgery. The patterns of recurrence and the detailed descriptions are shown in Table 1. Among the recurrent patients, 33.3% (7/21) were reoperated, and 1 patient suffered relapse once again after reoperation (Figure 2A–E).

**TABLE 1:**
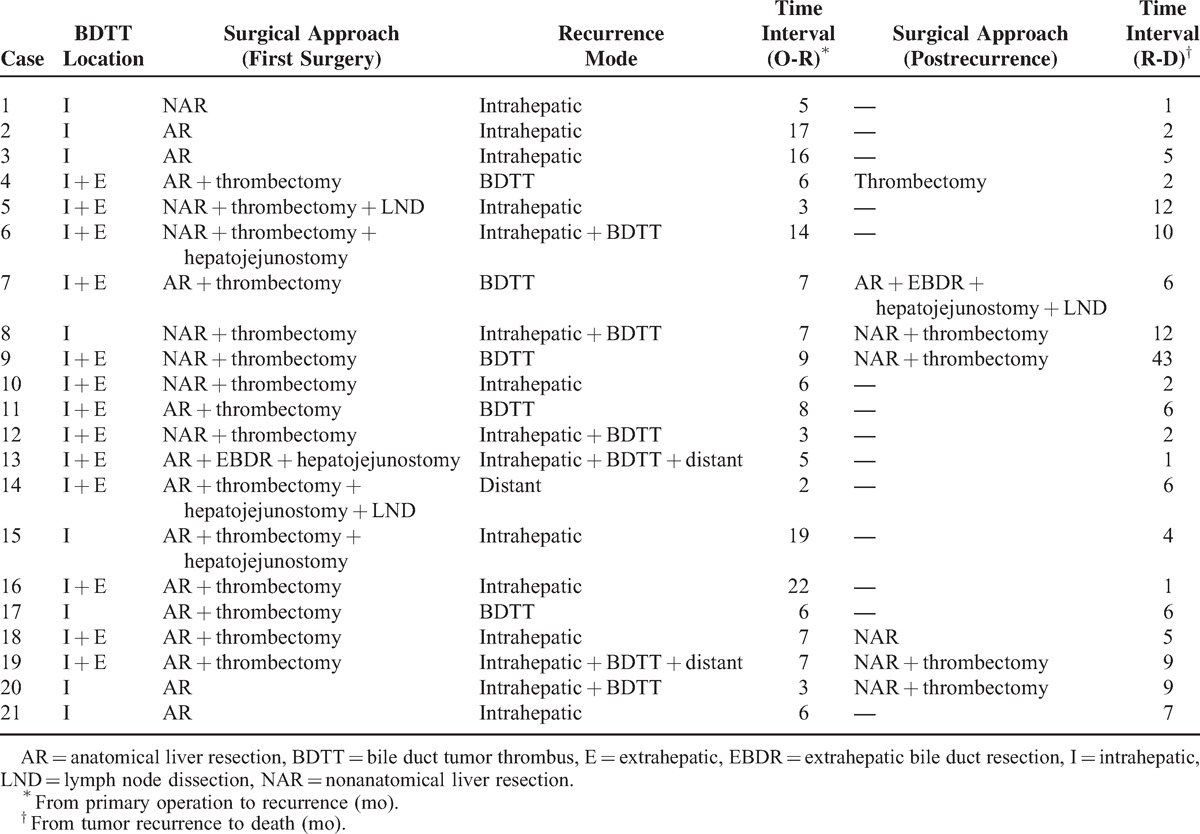
Management and Survival of the 21 Recurred Patients

### Risk Factors of Tumor Recurrence

**FIGURE 3:**
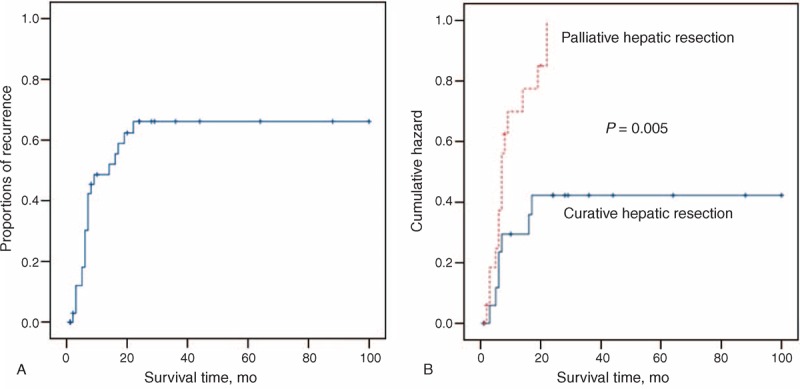
Cumulative recurrence curve of HCC and BDTT patients. (A) The cumulative recurrence rates of the 37 patients with HCC and BDTT. (B) The overall recurrence rate of the 37 patients with HCC and BDTT according to surgical curability. BDTT = bile duct tumor thrombus, HCC = hepatocellular carcinoma.

As shown in Figure 3A, cumulative recurrence rates were 43.2% at 1 year and 56.8% at 2 years. After recurrence, the median survival time was 9.0 months in the group of patients undergoing reoperation, which was significantly longer than that in the conservative management group (4.0 months; *P* = 0.042). To determine the independent risk factors involved in the recurrence of HCC with BDTT, univariate binary logistic regression analysis was performed first. Data showed that tumor histological grade (*P* = 0.044) and surgical curability (*P* = 0.015) were associated with tumor recurrence after surgery. Sex, age, HBsAg, cirrhosis, serum AFP level, tumor size, tumor number, satellite nodules, tumor stage, vascular invasion, tumor metastasis, and type of resection (AR or NAR) did not influence recurrence (Table 2). Potential risk factors were further examined using multivariate binary logistic regression analysis. The results revealed that surgical curability was the only independent predictor of tumor recurrence (relative risk [RR]: 5.099; 95% confidence interval [CI]: 1.128–23.052; *P* = 0.034). As shown in Figure 3B, the overall postoperative recurrence rate of curative resection group was 36.8%, which was significantly lower than that of the palliative resection group (77.8%; *P* = 0.005).

**TABLE 2:**
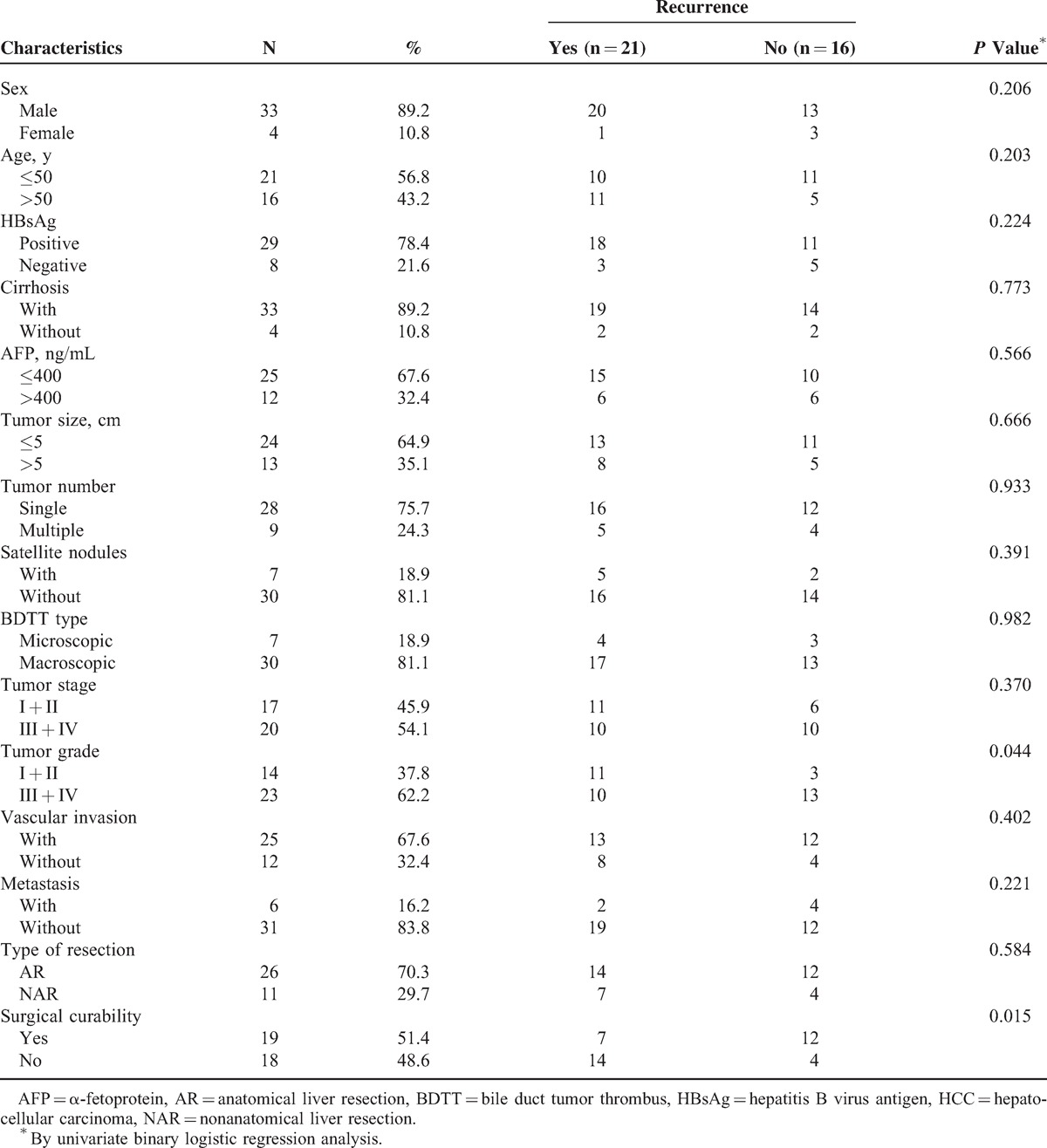
Univariate Analysis of Clinicopathological Features Associated With Recurrence After Surgery in HCC With BDTT

### Patient Survival and Prognostic Factors

**FIGURE 4:**
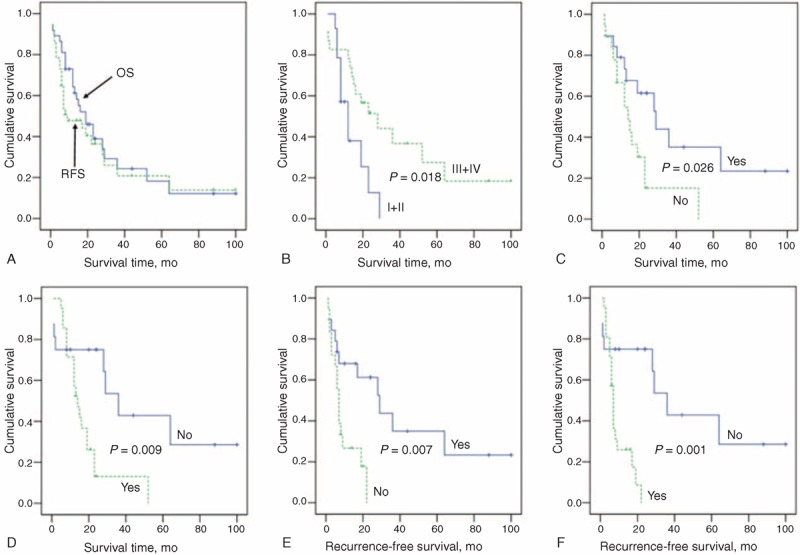
Postoperative OS and RFS curves of HCC and BDTT patients. (A) Postoperative OS and RFS of the 37 patients with HCC and BDTT. (B–D) The comparison of OS rates of the 37 patients with HCC and BDTT according to histological grade (B), surgical curability (C), and tumor recurrence (D). (E and F) The comparison of RFS rates of the 37 patients with HCC and BDTT according to surgical curability (E) and tumor recurrence (F). BDTT = bile duct tumor thrombus, HCC = hepatocellular carcinoma, OS, overall survival, RFS, recurrence-free survival.

The follow-up span was 1 to 100 months and the median follow-up time was 19 months. Overall survival (OS) rates were 64.2% at 1 year, 38.9% at 2 years, and 24.3% at 3 years (Figure 4A). In addition, recurrence-free survival (RFS) rates at 1, 2, and 3 years were 47.8%, 36.4%, and 20.8% (Figure 4A), respectively. On univariate analysis, high histological grade, curative hepatic resection, and no tumor recurrence were associated with better OS after initial surgery (*P* < 0.05; Table 3; Figure 4B–D), while curative hepatic resection and no tumor recurrence were associated with prolonged RFS (*P* < 0.05; Table 3; Figure 4E and F). Multivariate analysis revealed that surgical curability and tumor recurrence were independent prognostic factors for both OS and RFS (*P* < 0.05; Table 4).

**TABLE 3:**
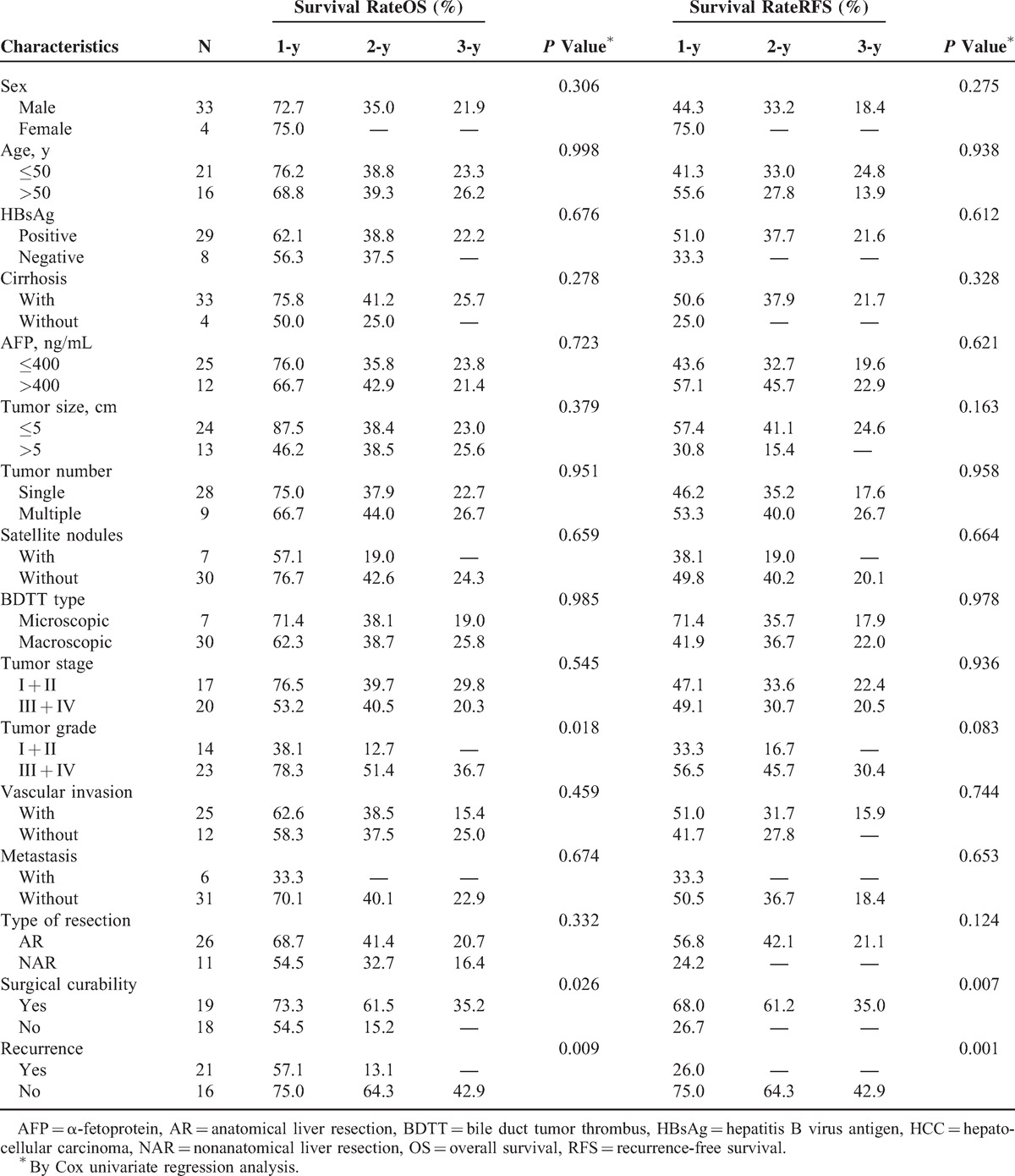
Clinicopathological Features and Survival Rates of the 37 HCC Patients With BDTT

**TABLE 4:**
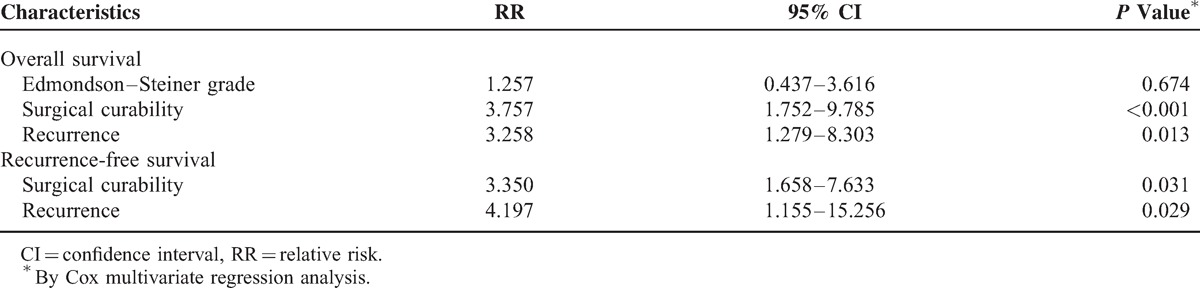
Multivariate Analysis of Risk Factors Affecting Overall Survival and Recurrence-Free Survival

As shown in Figure 4C, OS rates at 1, 2, and 3 years of curative resection group were 73.3%, 61.5%, and 35.2%, respectively, which were significantly higher than those of the palliative resection group (54.5%, 15.2%, and 0%, respectively; *P* = 0.026). Similarly, the 1-, 2-, and 3-year RFS rates of curative resection group were 68.0%, 61.2%, and 35.0%, respectively, which were significantly higher than those of the palliative resection group (26.7%, 0%, and 0% respectively; Figure 4E; *P* = 0.007). The median OS time was also longer in the AR group (34.5 ± 8.0 months) compared with that in the NAR group (24.7 ± 8.0 months), but without statistical significance (*P* = 0.332). In addition, the results of the log-rank test showed that tumor recurrence had a significant negative influence on the long-term outcome of patients with HCC and BDTT (Figure 4D and F).

## DISCUSSION

The diagnosis and treatment of HCC with BDTT have progressed remarkably in recent years. An increasing number of studies have shown that surgical treatment may offer these patients a chance of cure. However, the total recurrence rate after curative resection is still high even in early stage patients. Qin et al^8^ reported that the intrahepatic tumor recurrence within 1 year after operation was found in 50% of patients. Ikenaga et al^10^ showed that HCC with bile duct invasion has an infiltrative nature and 53% of patients suffered recurrences in the remnant liver within 3 months after surgery. Shao et al^11^ reported that 70.3% of patients with BDTT experienced tumor recurrence within 1 year after surgery. In our series, 56.8% of patients recurred after surgery and most of them recurred within 12 months. Multivariate analysis and log-rank test revealed that tumor recurrence had a significant negative influence on the long-term outcome of patients with HCC and BDTT, which is consistent with the results of other reports.^8,10,11^

Notably, our results showed that the recurred patients who underwent reoperation had significantly longer survival while those with conservative management had marginally prolonged survival. Similar results had been published previously by Peng et al,^7^ who found that repeated surgery yielded a good outcome for tumor recurrence in patients with HCC and BDTT. Therefore, we must notify that HCC with BDTT has a high risk of early recurrence even after radical surgery, causing poor prognosis. Prevention and effective treatment of postoperative recurrence are useful to improve the OS rate after operation. Hence, more frequent follow-up of these patients should be considered to promote earlier detection of recurrence.

Some authors have concluded that early intrahepatic recurrences of HCC appear to arise mainly from intrahepatic metastases of the primary tumor, whereas late recurrences are more likely to be multicentric occurrence of new tumors. In the past, spreading via the blood system is considered the main route of intrahepatic metastasis.^16,25^ Noda et al^15^ showed that portal or hepatic vein tumor thrombosis was the only significant determinant of poor prognosis in patients with HCC and BDTT. Likewise, Poon et al^16^ reported that venous invasion was independent risk factor for early intrahepatic recurrence after resection of HCC. The findings of Toyosaka et al^25^ suggest that tumor spread in HCC progresses from capsular invasion to intrahepatic invasion and the portal vein may act as an efferent tumor vessel. However, many studies found that patients with HCC and BDTT also suffered early recurrences in the remnant liver without evidence of major vascular invasion. Moreover, vascular invasion did not have an impact on tumor recurrence in patients with HCC and BDTT, while high recurrence rate was also found in those without vascular invasion.^8,10,11^ In the present study, though with a relatively high incidence, vascular invasion is not a risk factor for tumor recurrence and poor prognosis. As a consequence, it was noteworthy that patients with HCC and BDTT also have a high rate of BDTT recurrence. Peng et al^7^ reported that BDTT recurred in 37.5% of patients after surgery, and suggest that the unresected primary tumor might well be the source of the BDTT recurrence. Shao et al^11^ found that 25.9% of these patients experienced BDTT recurrence, and they speculate that tumor cells invading the neighboring biliary system may be another route of intrahepatic metastasis. Our data are consistent with the results of other studies in showing that as high as 52.3% of patients experienced BDTT recurrence, and 1 patient suffered BDTT relapse once again after rethrombectomy. Thus, these studies indicate that tumor migration through the biliary tract may be one of the main causes of HCC metastasis.

In addition, our data revealed that surgical curability and tumor recurrence were independent prognostic factors for both OS and RFS, while surgical curability was the only independent predictor of tumor recurrence. Therefore, curative hepatic resection can effectively reduce the recurrence rate and can significantly improve the outcome of patients with HCC and BDTT. Previously, complete surgical excision of the primary liver tumor with thrombectomy through a choledochotomy is a rational technique for curative resection of HCC with BDTT. It is believed that BDTT rarely invades the walls of the large bile ducts around the hepatic hilus, and BDTT can be easily peeled off. The indication for concurrent bile duct resection was macroscopic tumor invasion of the large bile ducts.^3–5,12,26^ Some authors have also concluded that there were no significant differences in the survival rates between patients who underwent bile duct resection and those who did not.^3,4^ On the other hand, however, some observations have shown that tumor thrombectomy through a choledochotomy is contraindicated for surgical treatment of HCC with BDTT, as this procedure might cause tumor recurrence at the site of the choledochotomy probably due to intraoperative iatrogenic implantation.^27,28^ The question therefore in controversy is whether tumor thrombectomy through a choledochotomy is appropriate for these patients. The key to solve the problem is to define whether the tumor thrombi can directly invade the bile duct wall.

**FIGURE 5:**
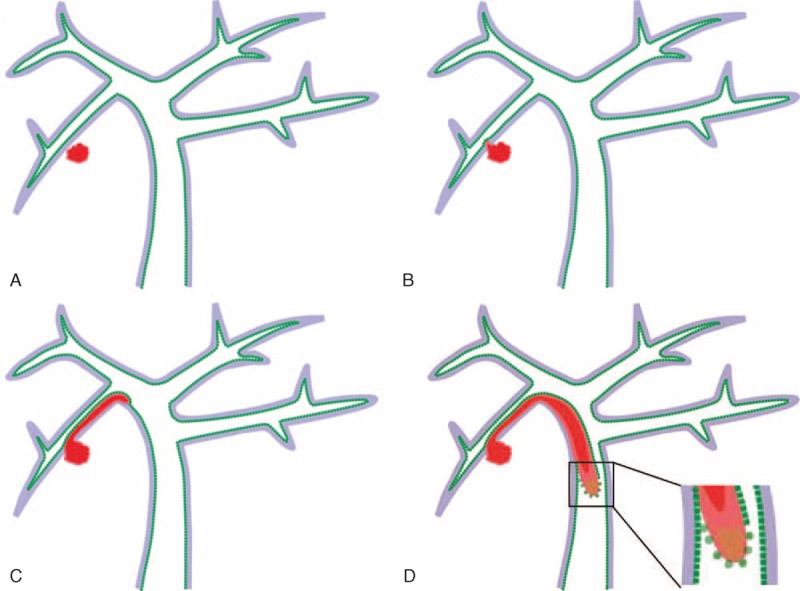
A schematic showing the mode of invasion and metastasis of BDTT. (A) Primary tumor lies inside liver parenchyma. (B) Tumor cells invading the submucosa of adjacent small bile ducts. (C) Tumor cells disseminate along the biliary route. (D) Tumor cells tend to intrude into the lumen of bile duct and form BDTT. BDTT = bile duct tumor thrombus.

Our present results showed that in 16 patients who underwent thrombectomy, bile duct invasion was confirmed pathologically in 10 patients, while in 14 patients who underwent concurrent bile duct resection, 10 patients had histologically confirmed bile duct invasion. Tumor recurrence rate was significantly higher in patients who underwent thrombectomy than in patients with concurrent bile duct resection. In addition, the tumor recurrence rate was significantly lower in the curative resection group than in the palliative resection group, but there were no significant differences between patients with AR and NAR. That is to say, up to two-thirds of patients with HCC and BDTT had histopathological evidence of direct tumor invasion into the bile duct wall preoperatively or intraoperatively. Based on histological and ultrastructural findings, we speculated that tumor cells first invade the subepithelium of adjacent small bile duct, and then they grow continuously along the bile duct wall to extrahepatic bile duct (Figure 5). However, it remains unknown in which level the tumor cells break through the bile duct wall and cause a thrombus in the lumen of bile duct. Therefore, although AR remains the gold standard in the treatment of HCC, AR alone cannot achieve complete tumor clearance in the vast majority of patients with HCC and BDTT and is unable to reduce the rate of tumor recurrence. Based on this, we considered that concurrent bile duct resection was required in most patients to achieve curative hepatic resection and get better survival. Similarly, Wang et al^28^ have reported that it was necessary to perform major hepatic resection with removal of the extrahepatic bile duct to improve survival and therapeutic results.

In conclusion, patients with HCC and BDTT have a relatively high rate of early recurrence after surgery. The prognosis of this type of HCC is extremely dismal, but the survival can be improved if complete tumor clearance is achieved. Therefore, AR plus extended bile duct resection would be a more appropriate strategy than AR alone for preventing tumor recurrence and improving the survival rate in this subset of patients. However, further studies with a larger number of patients are needed to draw the final conclusion.
